# Rapid reduction of depressive symptoms with minimal dissociation: results from the KET01-02 and KET01-03 trials with oral prolonged-release (PR) ketamine KET01

**DOI:** 10.1192/j.eurpsy.2024.220

**Published:** 2024-08-27

**Authors:** C. zu Eulenburg, E. Papanastasiou, K. Schmid, A. Damyanova, A. Glas, C. Strote, L. Arvastson, H. Å. Eriksson

**Affiliations:** ^1^HMNC Brain Health, Munich, Germany; ^2^Develco Pharma, Pratteln, Switzerland

## Abstract

**Introduction:**

Current ketamine-based therapies for treatment-resistant depression (TRD) often induce dissociative effects. A novel oral PR ketamine formulation (KET01) results in a low and delayed peak concentration of ketamine, high hydroxynorketamine concentration, and is associated with limited dissociative properties.

**Objectives:**

To investigate efficacy, safety, and pharmacokinetics of KET01 in TRD.

**Methods:**

KET01-02 was a randomized, double-blind phase 2 trial in outpatients with TRD comparing adjunct 120 mg (n=42) or 240 mg (n=40) oral KET01 once-daily for 3 weeks to placebo (PBO, n=40). The primary endpoint was change from baseline in the MADRS mean score on Day 21. KET01-03 was a randomized, double-blind, cross-over phase I trial in 26 healthy volunteers comparing single doses of 240 mg oral KET01 and 84 mg an approved intranasal formulation of eketamine. The primary endpoint was maximum change of Clinician-Administered Dissociative States Scale (CADSS) score from baseline.

**Results:**

KET01-03 trial; the mean (±SD) maximum change of CADSS score within 24 hours after dosing was 29.6±12.5 for intranasal eketamine and 0.7±1.7 for KET01 (p<0.00000000001). KET01-02 trial; no differences in CADSS score (range: 0.2 to 1.3), and heart rate and blood pressure were observed between the groups on Day 1 and beyond. 10%, 12%, and 15% of patients in the PBO, 120 mg/day, and 240 mg/day KET01 groups, respectively had CADSS score >4 and increase from baseline. At 7 hours post first KET01 dose (240 mg), plasma concentration of ketamine (38.7±27.0 ng/ml) was lower than its metabolites norketamine (267.5±81.6 ng/ml) and hydroxynorketamine (190.2±85.5 ng/ml). 240 mg/day KET01 induced clinically relevant reduction from baseline in MADRS score already within the first 7 hours of treatment (-7.65; Δ vs PBO: -2.22, n.s.), with a statistically significant separation on Day 4 (-10.02; Δ vs PBO: -3.66, p=0.020) and Day 7 (-12.21; Δ vs PBO: -3.95, p=0.042). MADRS score decrease was sustained throughout Day 21 (-13.15; Δ vs PBO: -1.82, n.s.), and during 4-week follow-up (-12.51; Δ vs PBO: -3.35, n.s.). Treatment-emergent adverse events occurred in 47.5%, 50.0%, and 62.5% of patients in the PBO, 120 mg/day, and 240 mg/day KET01 group, respectively.

**Conclusions:**

Oral 240 mg/day KET01 induces a rapid, and clinically relevant reduction of depressive symptoms with only minimal signs of dissociation, potentially due to lower ketamine levels and increased norketamine and hydroxynorketamine levels compared to intravenous administration. Our results suggest that KET01 may be an efficacious and safe take-at-home adjunct treatment for TRD.

**Disclosure of Interest:**

C. zu Eulenburg Employee of: HMNC Brain Health, E. Papanastasiou Employee of: HMNC Brain Health, K. Schmid Employee of: Develco Pharma, A. Damyanova Employee of: HMNC Brain Health, A. Glas Employee of: HMNC Brain Health, C. Strote Employee of: HMNC Brain Health, L. Arvastson Employee of: HMNC Brain Health, H. Eriksson Employee of: HMNC Brain Health

